# Human values and physical activity before and during COVID-19 restrictions in Hungary

**DOI:** 10.1038/s41598-026-41883-8

**Published:** 2026-02-28

**Authors:** Gergely Csurilla, Imre Fertő, Zsófia Benedek, József Fogarasi, Zoltán Bakucs

**Affiliations:** 1 Institute of Economics, ELTE Centre for Economic and Regional Studies, 1097 Budapest, Tóth Kálmán u. 4, Budapest, Hungary; 2 Sport Economics and Decision Making Research Centre, Hungarian University of Sports Science, Budapest, Hungary; 3 Corvinus University of Budapest, Budapest, Hungary; 4 Department of Economics, Czech University of Life Sciences, Prague, Czech Republic; 5 Óbuda University, Budapest, Hungary; 6 Partium Chrisitan Univeristy, Oradea, Romania

**Keywords:** Psychographic segmentation, Schwartz values, Physical activity, Natural experiment, Behaviour change, Health care, Psychology, Psychology, Risk factors

## Abstract

Physical inactivity is a major public health challenge in Hungary. Drawing on Schwartz’s Theory of Basic Human Values, we exploit a natural experiment created by temporary population-wide restrictions to examine how value orientations relate to physical activity across contrasting contexts. In a nationally representative survey of 1,031 adults, respondents reported frequency of structured exercise training (SET) and light daily physical activity (LDPA) for the period before the restrictions and during them. Generalized ordered logistic models linked activity categories to self-transcendence, conservation, openness to change, and self-enhancement, controlling for sociodemographic and health factors. Self-transcendence predicted higher participation in both SET and LDPA under usual conditions; during restrictions, its association with SET attenuated, whereas the link with LDPA remained robust. Conservation values consistently predicted lower SET, with avoidance intensifying under constraints; associations with LDPA were weaker. Openness to change and self-enhancement showed no independent effects after adjustment. Age, gender, education, self-rated health, BMI, smoking and alcohol use were associated with activity. Findings indicate that values shape activity differently depending on context: self-transcendence is associated with greater persistence in daily activity, whereas conservation corresponds to declines in structured exercise. Aligning interventions with motivational profiles may improve adherence when opportunities to be active are disrupted.

## Introduction

Physical inactivity remains one of the most serious public health challenges of our time. Globally, insufficient physical activity is associated with increased risks of cardiovascular disease, hypertension, and type 2 diabetes, and represents a leading contributor to premature mortality, and avoidable health-care expenditures^1^. In Hungary, physical inactivity is particularly widespread: in 2017, about 67% of adults were classified as physically inactive, only a modest improvement from the peak inactivity level of 77% observed in 2009^2^. Even relatively small population-level increases in regular physical activity have been shown to generate substantial health and economic benefits^3^. These patterns are further reflected in Hungary’s comparatively low life expectancy at birth (76.9 years), which remains well below the European Union average of 81.5 years^4^. Together, these figures underscore the urgency of understanding why some individuals maintain physically active lifestyles while others reduce or abandon physical activity.

Much of the existing literature on sport participation and physical activity describes participation patterns using sociodemographic characteristics such as age, gender, and education^5^. Although these factors help describe participation patterns, they account for only a limited share of behavioural variation. Individuals with similar observable characteristics often differ substantially in their exercise habits, suggesting that deeper motivational factors play an important role^6^. As a result, sport science research has increasingly turned toward psychographic approaches that emphasize underlying motivations and values, rather than demographics alone^6,7^. Prior work also indicates that implementing individually tailored physical activity prescription in routine care depends on supportive implementation strategies and organisational resources^8^.

**Fig. 1:**
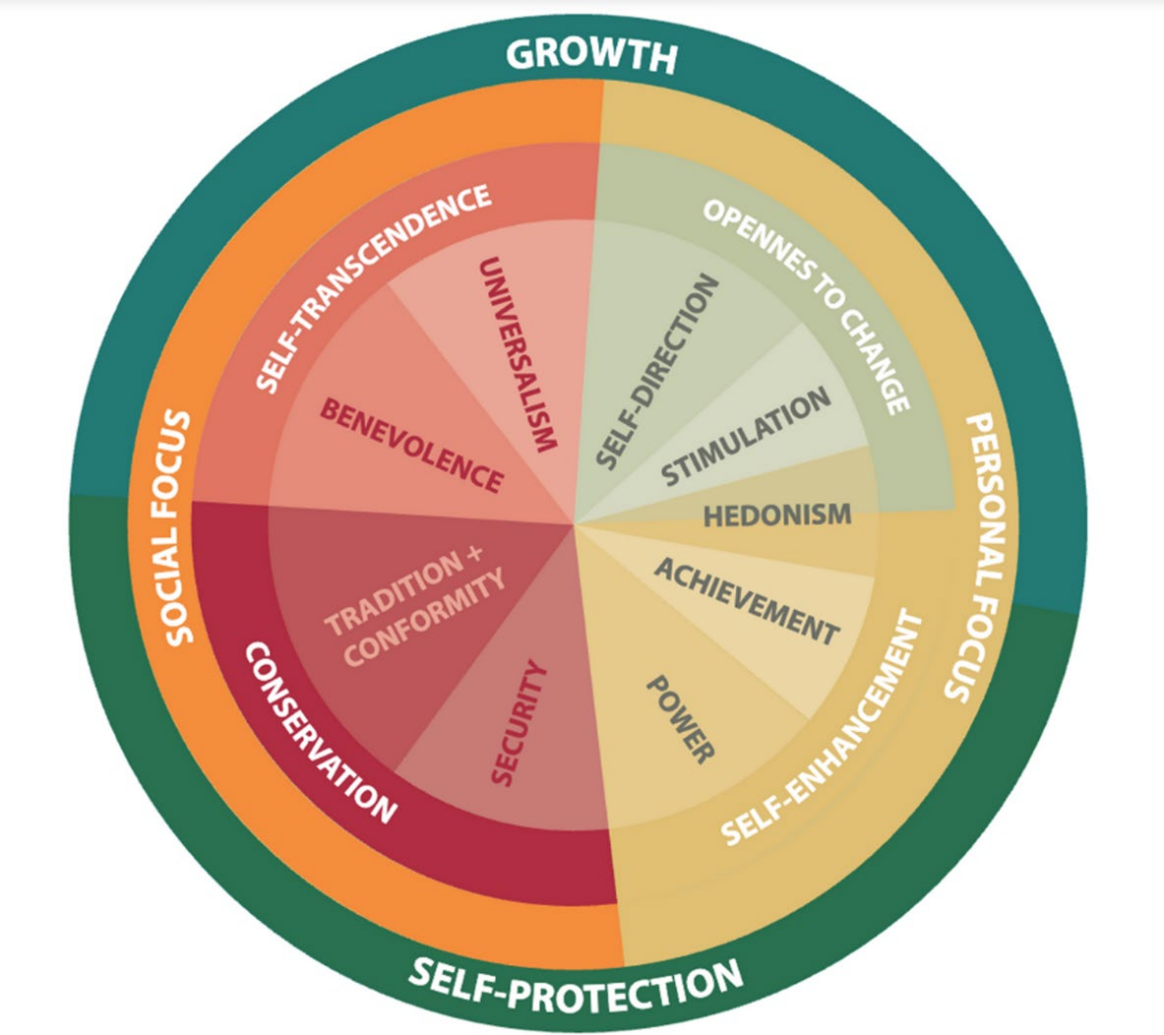
Schwartz’s^9^ circumplex model of basic human values.

In this study, we focus on basic human values as conceptualized in Schwartz’s Theory of Basic Human Values (TBHV)^9^. Values represent broad, relatively stable motivational goals that guide behavior across domains and over time. Unlike proximal psychological constructs such as attitudes or intentions, which are often situation-specific and sensitive to short-term contextual changes, values reflect enduring priorities – such as security, openness to novelty, concern for others, or personal achievement – that shape behavioural choices across contexts. Because values operate at a general motivational level, they can shape multiple behaviours, including health behaviours, across situations. Accordingly, while attitudes and intentions are important predictors of short-run exercise choices, values are particularly useful for explaining persistence and adaptation across contexts. TBHV organizes values into a circumplex structure that is commonly summarized into four higher-order dimensions (see Fig. 1): Self-Transcendence, Conservation, Openness to Change, and Self-Enhancement^9^, and has been used to examine value behaviour links in everyday activity, including physical activity^10^. These dimensions capture fundamental motivational tensions, including concern for others versus self-interest and stability versus novelty seeking.

Empirical evidence on how value orientations relate to physical activity is still relatively limited and suggests that different values may motivate activity for different people. In real-time reports, physical activity episodes have been linked to multiple value states, including achievement, power, hedonism, and personal security^10^. Cross-cultural work further indicates that certain self-transcendence facets (e.g., universalism-nature) and openness-to-change values (e.g., stimulation) can be positively related to physical activity in some settings, whereas conservation-related values (e.g., security) tend to be negatively related^11^. At the same time, these associations appear to be context-dependent, suggesting that environmental conditions may moderate the relationship between values and physical activity behavior^11^.

A key advantage of a value-based approach is that it allows for psychographic segmentation of the population according to intrinsic motivational orientations rather than observable characteristics alone^6,7^. This is particularly relevant for understanding behavioral persistence. Values are expected to shape how individuals respond when habitual routines are disrupted and opportunities for physical activity change. If values function as stable motivational anchors, they should be especially informative about who maintains activity when opportunities are disrupted.

The COVID-19 pandemic provides a unique opportunity to examine this issue. Population-wide restrictions introduced during the pandemic sharply reduced opportunities for structured exercise training (SET), such as gym-based workouts or organized sports, while leaving lighter forms of daily physical activity (LDPA), including walking or household activities, relatively less constrained^12,13^. In this sense, the pandemic can be viewed as a natural “stress test” of value-guided physical activity behaviour. Population-wide restrictions introduced during the pandemic coincided with low compliance with activity recommendations and high screen time in European samples, and comparisons with pre-lockdown periods indicate decreases in physical activity in some groups^12,13^. For example, Luciano et al.^13^ observed decreased physical activity alongside increases in sitting and sleep time among Italian medical students from pre- to during lockdown. Other studies have highlighted adverse psychosocial consequences of reduced activity, including increased insomnia and loneliness, particularly among women^14^, as well as longer-term declines in physical fitness following prolonged restrictions^15^.

Despite this growing literature, relatively little is known about how changes in physical activity during the pandemic differed across motivational profiles rooted in basic human values. Most studies focus on average effects or demographic subgroups, offering limited insight into psychological heterogeneity. Moreover, few analyses explicitly distinguish between structured exercise training and light daily physical activity, even though organised/structured and non-organised forms of activity differ in their resource requirements and show different longitudinal associations with overall activity patterns^16,17^. Longitudinal evidence further suggests that individuals follow heterogeneous physical activity trajectories during and after lockdowns, reinforcing the importance of modelling heterogeneity rather than average trends^18^.

The primary objective of this study is therefore to examine how basic human values are associated with participation in structured exercise training (SET) and light daily physical activity (LDPA), and how these associations differ between normal conditions and a period of pandemic-related restrictions. Using a nationally representative sample of Hungarian adults, we analyze the relationship between value orientations and ordered categories of SET and LDPA frequency before and during the COVID-19 pandemic.

This study contributes to the literature in three ways. First, it provides systematic evidence on how Schwartz’s value orientations relate to different types of physical activity in a representative adult population. Second, by exploiting the COVID-19 pandemic as a period of externally imposed constraints, it sheds light on the resilience and vulnerability of value-based motivational profiles under adverse conditions. Third, it offers insights relevant for the design of value-congruent physical activity interventions aimed at sustaining activity when conventional exercise opportunities are disrupted.

## Data and methodology

### Conceptual framework and value measurement

Our aim is to capture relatively stable, trans-situational motivations that help describe heterogeneity in physical activity across contexts. There are several frameworks available. Proximal belief-based models (e.g., Health Belief Model, Theory of Planned Behaviour) and stress–coping perspectives (e.g., Lazarus and Folkman^19^ are informative for situation-specific appraisals, intentions, and perceived control, but our focus is on broader motivational orientations that may generalize across behaviours and conditions.

To that end, we adopt Schwartz’s Theory of Basic Human Values, a framework that summarizes universal value priorities and has demonstrated cross-cultural measurement invariance^9,10,20^. Value priorities were measured using the 21-item Portrait Values Questionnaire (PVQ-21; the European Social Surve (ESS) Human Values Scale logic), which presents 21 short portraits describing a person’s goals and aspirations. Respondents indicated how similar each portrait was to themselves on a six-point scale (1 = not like me at all; 6 = very much like me), coded such that higher scores reflect stronger endorsement. Following the PVQ-21 scoring protocol, the 21 items yield ten basic values (universalism is measured with three items; all other values with two items), computed as the mean of their constituent items. We deliberately relied on non-ipsatised (raw) value scores to preserve between-person differences in overall value endorsement; ipsatisation, although sometimes used to address response-style heterogeneity, entails non-trivial trade-offs for regression inference^21^. As a descriptive check addressing response-style tendencies, Appendix Table 7 and 8 report Spearman correlations between the four physical activity measures and ipsatised value priorities (the 10 basic values in 7 and the four higher-order value domains in 8). Ipsatisation was implemented by subtracting each respondent’s mean PVQ-21 rating (across the 21 items) from each value index.

TBHV specifies ten basic values arranged in a circumplex (“value wheel”) and commonly summarized into four higher-order dimensions (see Fig. 1): Openness to Change (self-direction, stimulation; hedonism sits at the border per scoring conventions), Self-Enhancement (achievement, power; hedonism per scoring), Conservation (security, tradition, conformity), and Self-Transcendence (benevolence, universalism). A natural question is why we opt for a four-group solution instead of modelling the original ten TBHV values separately. Our choice follows the hierarchical nature of the Schwartz circumplex: the ten basic values form a motivational continuum and cluster into broader oppositions, while the four higher-order domains capture the two fundamental conflicts – Openness to Change versus Conservation, and Self-Enhancement versus Self-Transcendence – and have been shown to provide reliable, cross-culturally robust constructs^9,22^. From an analytical standpoint, working at the higher-order level offers a parsimonious representation of general value orientations, reduces redundancy among closely neighbouring values in the circumplex, and facilitates interpretation and comparability with prior work. Although multicollinearity diagnostics in our sample were reassuring (OLS VIFs ranged from 1.08 to 2.37), estimating models with all ten partially overlapping predictors would substantially increase model complexity with limited incremental information, potentially obscuring substantive interpretation and, in addition, jeopardies model convergence. A substantial body of work links TBHV to health-related behaviours, notably physical activity, underscoring its utility for modelling the motivational foundations of behaviour^10,11^.

### Data

Nationally representative quantitative data were obtained through structured telephone interviews conducted within a cross-sectional design, adhering to the STROBE recommendations for survey research^23^. Data were collected through a single questionnaire survey administered during the third wave of the COVID-19 pandemic in June 2021, which also included retrospective questions referring to the pre-pandemic period (i.e., the year 2019). Ethical approval was granted by the Ethical Committee of the ELTE Centre for Economic and Regional Studies (approval no. 1FŐIG/64/2024). All procedures were performed in accordance with relevant guidelines and regulations. Verbal informed consent was obtained from all participants prior to interviews.

The final sample (*N* = 1031) was stratified to ensure representativeness of Hungarian adults (age ≥ 18) across major sociodemographic dimensions – namely gender, age, educational attainment, settlement type, and regional distribution – according to benchmarks provided by the 2016 Hungarian Microcensus. A professional surveying agency was employed for this task. We operate with four dependent variables: SET and LDPA in two time periods, before the pandemic^1^ and during the pandemic. SET is defined as planned structured physical activity of at least moderate intensity, undertaken with the purpose of improving or maintaining physical fitness, such as cycling, gym workouts, or team sports. On the other hand, LDPA covers light physical activity performed during leisure or daily routines such as walking, gardening or hiking. Although SET and LDPA may conceptually overlap, LDPA was intended to capture light, non-structured activities distinct from structured exercise. The respondents reporting frequent SET (“daily” or “more than once per week”) uniformly selected the highest LDPA frequency category as well, suggesting that the LDPA item was likely interpreted by some participants as overall physical activity (including SET) rather than light activity specifically. In addition, including a dummy variable equal to 1 for high-SET frequency and 0 otherwise was highly statistically significant in the LDPA models. Thus, to avoid double counting and ceiling effects biasing the LDPA models, we excluded these frequent SET respondents from the LDPA analyses (*n* = 333, 34% of the full sample). We are aware of the subjectivity of this decision, as we later mention in the limitations of this research.^2^

Table 1 reports the distribution of respondents across three broad SET frequency categories – before and during COVID-19. Although the original questionnaire offered seven response options for SET frequency, we observed minimal variation among some adjacent categories, some of them containing less than 3% of the observations. By collapsing these into three groups (reflecting “never or infrequent,” “occasional,” and “regular” participation), we were able to more clearly distinguish between those who never trained, those exercising sporadically, and those engaging frequently. This aggregation was chosen to ensure adequate observations per category and to enhance the robustness of our analysis. While it reduces data granularity, the negligible differences between adjacent original categories suggest that little information was lost – a trade-off we deemed acceptable for a clearer and more reliable classification of training frequency. Prior to the pandemic, 48.6% of participants fell into the “never or infrequent” group, 18.2% were “occasional,” and 33.2% trained regularly. During COVID‐19, these proportions shifted slightly to 47.1%, 20.3%, and 32.6%, respectively.

**Table 1:** Descriptive statistics of dependent variables, Number of SET.

Categories	SET		SET COVID	
	*N*	Frequency	*N*	Frequency
Never, less frequently	501	48.59%	468	47.13%
Monthly, weekly	188	18.23%	209	20.27%
More than one per week, daily	342	33.17%	336	32.58%
Total	1031	100%	1031	100%

Table 2 shows analogous statistics for low-intensity LDPA among the 683 participants who did not engage in frequent SET. Again, the initial seven‐point scale displayed limited variation between some categories, so responses were merged into three meaningful levels – “never or infrequent,” “occasional,” and “regular” – to better capture differences in LDPA. Before COVID-19, 15.23% of this subsample reported never or infrequent LDPA, 25.62% were occasional, and 59.15% trained regularly. During the pandemic these proportions shifted slightly to 16.69% never or infrequent, 24.16% occasional, and 59.15% regular.

**Table 2:** Descriptive statistics of dependent variables, Number of Light Daily Physical Activities.

Categories	LDPA		LDPA COVID	
	*N*	Frequency	*N*	Frequency
Never, less frequently	104	15.23%	114	16.69%
Monthly/weekly	175	25.62%	165	24.16%
More than one per week. daily	404	59.15%	404	59.15%
Total	683	100%	683	100%

Table 3 summarizes the key characteristics of our explanatory variables for the 1,031 respondents. The average age is 51.9 years, with participants ranging from 18 to 91. In terms of gender, 43.4% identified as male and 56.6% as female. Although the survey allowed four options (male, female, other, rather not say), every participant chose one of the first two categories – an outcome that is fairly typical in Central-Eastern European contexts.

**Table 3:** Descriptive statistics of explanatory variables.

Variable	Categories	*N*	Frequency /Mean	SD	Min	Max
Age		1031	51.9	17.3	18	91
Gender		1031		-	-	-
	0: Male	448	43.40%	-	-	-
	1: Female	583	56.60%	-	-	-
Education		1031		-	-	-
	1: primary	153	14.80%	-	-	-
	2: vocational	288	27.90%	-	-	-
	3: secondary	361	35%	-	-	-
	4: higher education	229	22.20%	-	-	-
N_family_		1028	2.68	1.31	1	9
Budapest		1031	-	-	-	-
	0: otherwise	721	69.93%	-	-	-
	1: Budapest	310	30.06%	-	-	-
Self-transcendence		1022	5.14	0.65	1.5	6
Conservation		1009	4.9	0.71	1	6
Self-enhancement		1026	4.24	0.85	1	6
Openness		1029	4.59	0.74	1	6
BMI		1031		-	-	-
	1: <= 25	411	39.86%	-	-	-
	2: > 25 & < 30	361	35.01%	-	-	-
	3: >30	259	25.12%	-	-	-
Health		1030	-	-	-	-
	1: bad	28	2.71%	-	-	-
	2: below average	88	8.54%	-	-	-
	3: average	525	50.97%	-	-	-
	4: above average	252	24.46%	-	-	-
	5: excellent	137	13.30%	-	-	-
Alcohol		1031	-	-	-	-
	0: abstinent	546	52.95%	-	-	-
	1: consumes	485	47.04%	-	-	-
Tobacco		1021	-	-	-	-
	0: not smoking	725	71.1	-	-	-
	1: smoking	296	28.99%	-	-	-

Educational levels in our sample are well spread across the spectrum: about one in seven respondents completed only primary schooling (14.8%), just over a quarter hold vocational qualifications (27.9%), around a third have a full secondary diploma (35%), and roughly one in five earned a tertiary degree (22.2%). Household size averages 2.7 people (SD = 1.3), with most homes ranging from two to four members. Geographically, 30% of participants live in Budapest, while the remaining 70% are distributed across smaller cities and rural areas.

In terms of value priorities, Self-transcendence – which reflects concern for the welfare of others and the broader community – emerges as the strongest motivator in our sample. Openness to Change – capturing curiosity, novelty-seeking, and a desire for personal growth – also scores well above midscale, suggesting many participants are receptive to new experiences, including varied forms of physical activity. By contrast, Self-enhancement – the drive for achievement and status – registers lower, indicating that competitive or status‐driven motives may play a smaller role in this context. Conservation values – emphasizing security, tradition, and conformity – sit between these poles, hinting that stability and routine are also important but less dominant than communal and exploratory goals.

With respect to health and lifestyle, Body Mass Index categories show that 39.9% of respondents have a BMI of 25 or below, 35% fall between 25 and 30, and 25.1% exceed a BMI of 30. These numbers are in line with international statistics (Eurostat, 2021) placing Hungary at the fourth place with 60% of the population being overweight (BMI > = 25) on the list of highest percentage of overweight population within the EU. Self-rated health skews toward the middle: 51% report “average,” 24% “above average,” 13.3% “excellent,” 8.5% “below average,” and just 2.7% “bad.” Many respondents with elevated BMI nonetheless rate their health as better than average – a well-documented bias in self-assessment among individuals who are overweight – so it is crucial to include both objective (BMI) and subjective (self-rated health) measures in the model^24,25^. Regarding substance use, 47% of respondents consume alcohol and 29% are current smokers; the remaining 53% abstain from alcohol and 71% do not smoke.

### Empirical framework

To examine how different exercise habits are explained by individuals’ Schwartz human value profiles and how an external shock (proxied by the COVID-19 pandemic) alters these relationships, we apply the following empirical approach.

Let *SET*_*i*_ denote the SET score for individual *i*,* LDPA*_*i*_ the analogous LDPA score, *TCov*_*i*_ the SET score measured during the COVID period and *LDPACov*_*i*_ the LDPA score during the COVID period. We summarize all the non-Schwartz control variables – namely age, gender, education level, family size, residence, BMI, self‐rated health, alcohol consumption, and tobacco use – into a single vector ***X***_*i*_. In addition, we introduce four indicators for Schwartz‐profile membership (Self‐transcendence, Conservation, Self‐enhancement, and Openness). The regression equation is then written as

1 $${Y}_{i}={\beta}_{0}+{{X}_{i}}^{T}\beta+{\delta}_{1}{ST}_{i}+{\delta}_{2}{Con}_{i}+{\delta}_{3}{SE}_{i}+{\delta}_{4}{Ope}_{i}+{\epsilon}_{i}$$

where *Y*_*i*_ stands in turn for *SET*_*i*_, *LDPA*_*i*_, *SETCov*_*i*_ or *LDPACov*_*i*_, ${\beta}_{0}$ is the intercept, $\beta$ is the coefficient vector on the nine-dimensional ***X***_*i*_, and ${\delta}_{1}$, ${\delta}_{2}$, ${\delta}_{3}$ and ${\delta}_{4}$ capture the separate effects with respect to Self-transcendence, Conservation, Self-enhancement or Openness. The disturbance term ${\epsilon}_{i}$is assumed to be i.i.d. standard logistic, which implies the logit link for the cumulative probabilities.

With an ordinal outcome variable – such as a ranked measure of training intensity and frequency – the ordered logit model is typically the first-choice estimator. This model assumes that each explanatory variable has a constant effect across all cumulative splits of the ordinal scale, an assumption often referred to as “proportional odds” or “parallel lines”^26,27^. In practice, however, this constraint can be overly restrictive: if a given covariate influences transitions between lower categories differently than transitions between higher categories, enforcing proportional odds leads to biased estimates and spurious inferences.

To diagnose this issue, we first estimated a standard ordered logit model and then applied the Brant test, which evaluates whether the coefficients estimated at each threshold of the ordinal outcome are statistically indistinguishable^28^. Rejection of the proportional odds hypothesis indicates that at least some predictors violate the parallel-lines assumption. In our dataset – where the vector of controls includes demographics (age, gender, education, family size, residence), health measures (BMI, self‐rated health), and behavioural indicators (alcohol and tobacco use) – the overall Brant test from the baseline ordered logit models were significant, indicating violation of the proportional odds assumption.

Generalized ordered logit model, may be applied if the parallel regression assumption fails^26,29^. In this framework, one can allow coefficients for certain variables to differ at each cumulative split, while preserving proportional odds constraints for those covariates that satisfy the parallel-lines test. Accordingly, we fitted a generalized ordered logit model that relaxes the parallel‐lines constraint only for the covariates that failed the variable‐specific Brant tests, while keeping the remaining controls constrained. By relaxing the uniformity restriction only where necessary, the generalized approach yields unbiased and more efficient estimates compared to a standard ordered logit when proportionality is untenable. The p-values reported in Table 4 refer to the post‐estimation Brant test from the fitted generalized ordered logit model, which assess whether the proportional‐odds constraints retained in the final specification are supported; thus, non‐significant p-values indicate that the parallel‐odds constraints are not rejected. Empirical comparisons consistently show that models fitted via generalized ordered logit outperform the constrained ordered logit in both goodness‐of‐fit and predictive accuracy whenever the Brant test is violated^26,29,30^.

**Table 4:** Generalized ordered logit estimates (gamma specification).

Variable	SET	SET COVID	LDPA	LDPA COVID
Age	-0.025***	-0.020***	-0.004	0.003
Gender	-0.495***	-0.422***	0.274	0.315*
Education	0.378***	0.265***	-0.012	0.059
N_family_	-0.064	-0.079	0.091	0.084
Budapest	0.048	-0.091	0.477***	0.413**
BMI	-0.201**	-0.213**	0.005	0.143
Health	0.305***	0.331***	0.439***	0.592***
Alcohol	0.323**	0.339**	-0.030	0.110
Tobacco	-0.483***	-0.347**	-0.118	-0.008
Self-transcendence	0.337***	0.193	0.438***	0.420***
Conservation	-0.455***	-0.390***	-0.049	-0.257*
Self-enhancement	0.165	0.139	-0.137	-0.142
Openness	0.173	0.137	0.184	0.172
Constant	-0.833	-0.127	-2.016*	-2.353**
*Statistics*				
*N*	984	984	651	651
*Pseudo R* ^*2*^	0.115	0.095	0.052	0.052
Wald test	0.00	0.00	0.000	0.000
AIC	1833.2	1899.6	1198.232	1208.741
BIC	1916.3	1982.7	1274.367	1284.876
Brant test	0.16	0.36	0.73	0.69

**Notes**: * *p* < 0.10, ** *p* < 0.05, *** *p* < 0.01.

The analysis is associational in nature. Although basic human values are generally considered stable over time, the cross-sectional design with retrospective reporting precludes causal inference. The findings should therefore be interpreted as documenting systematic associations between value orientations and physical activity behavior across contexts, rather than as causal effects of values on exercise participation.

## Results

Table 4 presents regression results examining how Schwartz’s basic human values are associated with participation in SET versus LDPA before and during the COVID-19 pandemic.

Prior to the pandemic, individuals with higher levels of self-transcendence – reflecting stronger emphasis on altruism, social connectedness, and concern for others – were more likely to report higher frequencies of both SET and LDPA. These associations are consistent with prior work linking Schwartz value priorities to physical activity patterns in some contexts (e.g.,^11^). During the COVID-19 restriction period, the relationship between self-transcendence and SET weakened and was no longer statistically significant. In contrast, the effect between self-transcendence and LDPA remained positive and statistically significant. Luciano et al.^13^ reported reductions in overall physical activity (including walking) during lockdown, suggesting that persistence of lower-intensity activity may vary by sample and context.

Conservation values – characterized by preferences for security, tradition, and conformity – were negatively associated with SET participation both before and during the pandemic. This pattern is consistent with research on values and everyday behavior (including security/conservation-related priorities) in relation to activity^10^ and with cross-cultural evidence connecting Schwartz values to physical activity patterns^11^. During the COVID-19 period, the magnitude of the negative impact between conservation values and SET increased, indicating a stronger inverse relationship under restrictive conditions. For LDPA, conservation values were weakly affected participation prior to the pandemic and showed a statistically significant negative association only during the COVID-19 period.

Openness to change values – capturing curiosity, novelty-seeking, and willingness to explore new activities – were not significantly associated with participation in SET or LDPA, either before or during the pandemic. This finding contrasts with results reported by Liang et al.^11^, who identified positive associations between openness to change and physical activity in specific cultural contexts. The present results indicate that, after controlling for sociodemographic and health-related variables, no independent relationship between openness to change and activity frequency was detected in the analysed samples.

Similarly, self-enhancement values – typically associated with achievement orientation, status seeking, and competitiveness – did not show statistically significant associations with SET or LDPA participation in any of the estimated models. This absence of significant associations is consistent with findings reported by Alexandris^6^, suggesting that achievement- or status-driven motivations may be less strongly related to regular exercise participation in non-competitive or recreational contexts.

Several control variables exhibited clear associations with SET participation. Older age and female gender were negatively associated with SET frequency in our sample. Higher educational attainment was positively associated with SET participation prior to the pandemic; however, the magnitude of this association declined during the COVID-19 period. Residence in Budapest was positively associated with LDPA participation, although this association weakened somewhat during the pandemic, potentially reflecting changes in mobility conditions. Better self-rated health had a positive impact on both SET and LDPA participation in both periods. Higher BMI and smoking status were negatively associated with SET participation, with similar effect sizes before and during the pandemic.

Table 5 reports predicted probabilities of SET and LDPA participation across selected levels of self-transcendence values. Before the pandemic, the probability of frequent SET participation increased with higher self-transcendence scores, rising from 18% at lower levels to 37% at higher levels. During the pandemic, this gradient was substantially attenuated for SET. In contrast, for LDPA, higher self-transcendence values were associated with higher probabilities of frequent participation both before and during the pandemic.

**Table 5:** Predicted probabilities of trainings by self-transcendence level.

Self-transcendenc					
		3	4	5	6
SET	never, less frequent	**0.65**	**0.57**	0.49	0.41
	monthly, weekly	*0.17*	*0.20*	*0.22*	*0.22*
	daily more than 1/week	*0.18*	*0.23*	0.30	0.37
LDPA	never, less frequent	0.27	*0.19*	*0.13*	*0.09*
	monthly, weekly	0.36	0.33	0.28	*0.23*
	daily more than 1/week	0.37	**0.47**	**0.58**	**0.68**
LDPA COVID	never, less frequent	0.25	*0.17*	*0.12*	*0.08*
	monthly, weekly	0.32	0.28	*0.22*	*0.17*
	daily more than 1/week	**0.44**	**0.55**	**0.66**	**0.76**

Table 6 presents predicted probabilities of SET participation across levels of conservation values. A pronounced inverse relationship is observed: individuals with higher conservation scores consistently exhibited lower probabilities of frequent SET participation. This pattern was present prior to the pandemic and became more pronounced during the COVID-19 period. For example, the predicted probability of frequent SET declined from 51% at lower conservation levels to 21% at the highest level during the pandemic.

**Table 6:** Predicted probabilities of trainings by conservation level.

Conservation					
		3	4	5	6
SET	never, less frequent	0.28	0.38	**0.49**	**0.60**
	monthly, weekly	*0.21*	*0.22*	*0.22*	*0.19*
	daily more than 1/week	**0.51**	**0.40**	0.30	*0.21*
SET COVID	never, less frequent	0.29	0.38	**0.47**	**0.57**
	monthly, weekly	*0.23*	*0.24*	*0.23*	*0.21*
	daily more than 1/week	0**.48**	0.39	0.30	*0.22*

## Conclusion

This study examined how basic human values are associated with engagement in different forms of physical activity – structured exercise training (SET) and light daily physical activity (LDPA) – and how these associations differ between normal conditions and the period of COVID-19–related restrictions. In line with previous research emphasizing psychographic heterogeneity in physical activity behaviour^5,18^, the results indicate that motivational orientations are systematically associated with participation patterns and that these associations vary across activity types and contexts.

Consistent with prior research linking Schwartz value priorities to physical activity in some settings (e.g.,^11^), individuals prioritizing self-transcendence values reported higher levels of both SET and LDPA under normal conditions. During the COVID-19 restriction period, however, the positive association between self-transcendence and SET was no longer observed, whereas the association with LDPA remained robust. This pattern suggests that, when opportunities for structured exercise are constrained, the association between self-transcendence and physical activity may be more evident for lower-intensity, everyday activities. Similar shifts toward lighter forms of physical activity during lockdowns have been documented in European populations^12–14^, and evidence of sustained post-lockdown fitness declines underscores the potential long-term relevance of interruptions to structured exercise^15^.

In contrast, conservation values – emphasizing security, tradition, and conformity – were consistently associated with lower participation in SET both before and during the pandemic. The negative association was stronger during the COVID-19 period, indicating that conservation-oriented individuals were less likely to engage in structured exercise under restrictive conditions. These findings are in line with work connecting Schwartz value priorities (including conservation/security) to physical activity participation patterns^11^. At the same time, associations between conservation values and LDPA were weaker, suggesting that routine, low-risk daily activities may be less incompatible with a preference for stability.

Values associated with openness to change and self-enhancement showed no independent associations with SET or LDPA participation in the estimated models. Contrary to findings that openness-to-change values can relate positively to physical activity in some contexts^11^, novelty-seeking and status-oriented motivations did not emerge as significant correlates once sociodemographic and health-related factors were accounted for. These results should be interpreted within the scope of the present analyses and do not imply that such values are unrelated to physical activity in general; rather, no independent associations were detected in this population and modelling framework.

The null associations for Openness to Change and Self-Enhancement are plausible in light of the strong opportunity constraints during the restriction period: novelty seeking or achievement-oriented motives may translate into physical activity only when relevant options (e.g., organised sport, facilities, varied settings) are available, and values typically predict behaviour more strongly when they are contextually activated and can be enacted. Moreover, using higher-order aggregates may attenuate effects if lower-order components operate in opposing directions (e.g., stimulation vs. self-direction within Openness to Change; achievement vs. power within Self-Enhancement), yielding a net coefficient close to zero at the domain level. Finally, LDPA results pertain to the non-high-SET analytic subsample by design; therefore, the absence of effects for these values in LDPA should not be generalized to individuals who engage in frequent structured exercise. Together, these considerations suggest that non-significant coefficients here are compatible with a context-dependent value–behaviour link rather than evidence against the motivational relevance of Openness or Self-Enhancement per se.

From a broader perspective, the findings highlight the importance of considering motivational heterogeneity when examining physical activity behaviour. The COVID-19 pandemic can be viewed as an external disruption that altered opportunities for physical activity, revealing differences in how value orientations are associated with engagement across activity types. This interpretation is consistent with evidence of heterogeneous physical activity trajectories observed in population-based studies during and after lockdown periods^18^, suggesting that individuals do not respond uniformly to external constraints.

The results also carry implications for the design of physical activity interventions. Aligning intervention strategies with underlying value orientations may enhance their relevance and acceptability. For example, community-oriented or socially framed activities may resonate more strongly with individuals high in self-transcendence, whereas familiar, low-risk, and home-based activities may be more suitable for individuals emphasizing conservation values. These implications should be regarded as suggestive and as directions for further empirical testing rather than prescriptive recommendations.

Several limitations should be considered when interpreting the findings. The study relies on self‑reported measures of physical activity and retrospective assessments of behaviour before and during the pandemic, which may be subject to recall bias and social desirability effects. The cross-sectional design cause for caution with retrospective causal inference. However, the possibility of bidirectional relationships between value orientations and physical activity behaviour is unlikely, since the values are the result of long-term evolution of personalities.

The exclusion of respondents with frequent SET participation from the LDPA models, might limit the national representativity of these results. However, we believe the bias introduced not excluding frequent SET participants would make the bias higher interpreting the motivations of people doing LDPA. To sum, our LDPA estimates should be interpreted as conservative and not directly generalizable to individuals with high levels of structured exercise.

Finally, although the sample is nationally representative of Hungary, cultural and institutional factors may limit the generalisability of the findings to other settings. Future research would benefit from longitudinal designs with repeated measurement of both values and physical activity, as well as cross-national comparisons, to further clarify how stable motivational orientations interact with changing opportunity structures. Experimental and longitudinal studies testing value-congruent intervention strategies may provide additional insight into how physical activity engagement can be supported across diverse populations and under challenging circumstances.

## Data Availability

The data that support the findings of this study are available from the corresponding author upon reasonable request.

## Appendix

**Table 7:** Spearman correlations between physical activity measures and ipsatized basic values.

	SET	SET COVID	LDPA	LDPA COVID	Power	Achieve-ment	Hedo-nism	Stimul-ation	Self-direction	Univer- salism	Bene-volence	Tradition	Confor-mity	Security
SET	1.000													
SET COVID	0.775***	1.000												
LDPA	0.254***	0.202***	1.000											
LDPA COVID	0.174***	0.241***	0.655***	1.000										
Power	0.059*	0.077**	-0.077**	-0.077**	1.000									
Achievement	0.119***	0.105***	-0.011	0.044	0.205***	1.000								
Hedonism	0.046	0.024	0.021	0.038	-0.039	-0.067**	1.000							
Stimulation	0.205***	0.182***	0.022	0.004	0.180***	0.121***	0.029	1.000						
Selfdirection	0.048	0.039	0.074**	0.094***	-0.225***	-0.057*	0.003	0.004	1.000					
Universalism	-0.034	-0.042	0.068**	0.072**	-0.475***	-0.339***	-0.115***	-0.316***	0.054*	1.000				
Benevolence	-0.008	-0.026	0.076**	0.047	-0.302***	-0.256***	-0.109***	-0.254***	-0.094***	0.270***	1.000			
Tradition	-0.185***	-0.159***	-0.031	-0.087***	-0.284***	-0.321***	-0.157***	-0.296***	-0.186***	0.020	0.060*	1.000		
Conformity	-0.147***	-0.121***	-0.066**	-0.075**	-0.275***	-0.266***	-0.206***	-0.399***	-0.142***	0.099***	0.094***	0.226***	1.000	
Security	-0.210***	-0.183***	-0.039	-0.006	-0.236***	-0.250***	-0.067**	-0.395***	-0.108***	0.148***	0.062**	0.068**	0.173***	1.000

** Notes**: Entries are Spearman rank correlations (ρ), computed pairwise using all available observations. * *p* < 0.10, ** *p* < 0.05, *** *p* < 0.01.

**Table 8:** Spearman correlations between physical activity measures and ipsatized higher-order value domains.

	SET	SET COVID	LDPA	LDPA COVID	Self-Transcendence	Conservation	Self-Enhancement	Openness to Change
SET	1.000							
SET COVID	0.775***	1.000						
LDPA	0.254***	0.202***	1.000					
LDPA COVID	0.174***	0.241***	0.655***	1.000				
Self-Transcendence	-0.029	-0.047	0.093***	0.072**	1.000			
Conservation	-0.271***	-0.235***	-0.071**	-0.098***	0.102***	1.000		
Self-Enhancement	0.148***	0.136***	-0.052*	-0.006	-0.560***	-0.577***	1.000	
Openness to Change	0.192***	0.159***	0.058*	0.075**	-0.321***	-0.603***	0.186***	1.000

** Notes**: Entries are Spearman rank correlations (ρ), computed pairwise using all available observations. * *p* < 0.10, ** *p* < 0.05, *** *p* < 0.01.

**Table 9:** Full-sample LDPA models with a frequent SET indicator and interactions with value dimensions.

Variable	LDPA	LDPA COVID
Age	-0.003	0.003
Gender	0.122	0.219
Education	-0.024	0.029
N_family_	0.097	0.075
Budapest	0.568***	0.394**
BMI	-0.008	0.119
Health	0.443***	0.550***
Alcohol	-0.132	0.035
Tobacco	-0.045	-0.026
Self-transcendence	0.418***	0.358**
Conservation	-0.107	-0.317**
Self-enhancement	-0.140	-0.153
Openness	0.173	0.142
SET x Self-transcendence	-0.020	0.877**
SET x Conservation	0.132	-0.889**
SET x Self-enhancement	-0.797**	0.154
SET x Openness	1.043**	0.063
Constant	-1.479	-1.229
*Statistics*		
*N*	984	984
*Pseudo R* ^*2*^	0.119	0.074
Wald test	0.000	0.000
AIC	1448.506	1607.796
BIC	1551.230	1720.303

** Notes**: * *p* < 0.10, ** *p* < 0.05, *** *p* < 0.01.

## Footnotes

1 To provide a time anchor to the respondent, the question presented was: “In the 12 month period before the COVID19 pandemic (March 2020) with which frequency were you doing Training/Easy Training”.

2 As a robustness check addressing the overlap between structured exercise and the LDPA item, we re-estimated the LDPA models on the full sample by introducing an indicator for frequent structured exercise participation and interacting it with the four value dimensions. Results are reported in Appendix Table 8 and support the same substantive interpretation.

## References

[1] World Health Organization. *Global Status Report on Physical Activity 2022*. 112 https://www.who.int/publications/i/item/9789240059153 (2022).

[2] Ács, P. et al. Comparative analysis of the economic burdens of physical inactivity in Hungary between 2005 and 2017. *BMC Public. Health*. **20**, 1174 (2020). 32799842 10.1186/s12889-020-08478-y PMC7429901

[3] Ács, P. et al. Economic and public health benefits: The result of increased regular physical activity. *Eur. J. Integr. Med.* **8**, 8–12 (2016).

[4] Eurostat. EU life expectancy estimated at 81.5 years in 2023. (2024).

[5] Taks, M. & Scheerder, J. Youth Sports Participation Styles and Market Segmentation Profiles: Evidence and Applications. *Eur. Sport Manage. Q.* **6**, 85–121 (2006).

[6] Alexandris, K. Segmenting recreational tennis players according to their involvement level: a psychographic profile based on constraints and motivation. *Managing Leisure*. **18**, 179–193 (2013).

[7] Mejova, Y. & Kalimeri, K. Effect of values and technology use on exercise: Implications for personalized behavior change interventions. in *Proceedings of the 27th ACM conference on user modeling, adaptation and personalization* 36–45Association for Computing Machinery, New York, NY, USA, 10.1145/3320435.3320451 (2019).

[8] Bouma, A. J. et al. Implementing Individually Tailored Prescription of Physical Activity in Routine Clinical Care: A Process Evaluation of the Physicians Implement Exercise = Medicine Project. *J. Phys. Activity Health*. **21**, 916–927 (2024). 10.1123/jpah.2023-0625 39084613

[9] Schwartz, S. H. Universals in the Content and Structure of Values: Theoretical Advances and Empirical Tests in 20 Countries. in *Advances in Experimental Social Psychology* vol. 25 1–65 (Elsevier, 1992).

[10] Skimina, E., Cieciuch, J., Schwartz, S. H., Davidov, E. & Algesheimer, R. Behavioral signatures of values in everyday behavior in retrospective and real-time self-reports. *Frontiers in Psychology* 10:281, (2019). 10.3389/fpsyg.2019.00281 PMC6401649 30873064

[11] Liang, Y., Rascle, O., Hanel, P. H. P., Yang, J. & Souchon, N. Values and physical activity among sports science students in France and China: a transcultural analysis. *Frontiers Psychology* 14:1304019, (2024). 10.3389/fpsyg.2023.1304019 PMC10794636 38239479

[12] Kovacs, V. A. et al. Physical activity, screen time and the COVID-19 school closures in Europe – An observational study in 10 countries. *Eur. J. Sport Sci.* **22**, 1094–1103 (2022). 33641633 10.1080/17461391.2021.1897166

[13] Luciano, F., Cenacchi, V., Vegro, V. & Pavei, G. COVID-19 lockdown: Physical activity, sedentary behaviour and sleep in Italian medicine students. *Eur. J. Sport Sci.* **21**, 1459–1468 (2021). 33108970 10.1080/17461391.2020.1842910

[14] Guerra-Balic, M. et al. Impact of COVID-19 lockdown on physical activity, insomnia, and loneliness among Spanish women and men. *Sci. Rep.* **13**, 2912 (2023). 36804465 10.1038/s41598-023-30173-2 PMC9941117

[15] Ripley-Gonzalez, J. W. et al. The long-term impact of the COVID-19 pandemic on physical fitness in young adults: a historical control study. *Sci. Rep.* **13**, 15430 (2023). 37723197 10.1038/s41598-023-42710-0 PMC10507106

[16] Luukkainen, N. M. et al. Longitudinal relationship between organised and non-organised physical activities and overall physical activity in children aged 3–11 years. *Eur. J. Sport Sci.* **24**, 1197–1206 (2024). 39054814 10.1002/ejsc.12172 PMC11295102

[17] da Silva, P. F. Physical activity patterns in adolescents: A longitudinal study. *Eur. J. Sport Sci.* **25**, e12239 (2025). 39716394 10.1002/ejsc.12239 PMC11667766

[18] Bu, F., Bone, J. K., Mitchell, J. J., Steptoe, A. & Fancourt, D. Longitudinal changes in physical activity during and after the first national lockdown due to the COVID-19 pandemic in England. *Sci. Rep.* **11**, 17723 (2021). 34475465 10.1038/s41598-021-97065-1 PMC8413348

[19] Lazarus, R. & Folkman, S. *Stress, Appraisal, and Coping* (Oxford University Press, 1984).

[20] Schwartz, S. H. & Cieciuch, J. Measuring the refined theory of individual values in 49 cultural groups: Psychometrics of the revised portrait value questionnaire. *Assessment* **29**, 1005–1019 (2022). 33682477 10.1177/1073191121998760 PMC9131418

[21] Rudnev, M. Caveats of non-ipsatization of basic values: A review of issues and a simulation study. *J. Res. Pers.* **93**, 104118 (2021).

[22] Schwartz, S. H. et al. Refining the theory of basic individual values. *J. Personal. Soc. Psychol.* **103**, 663–688 (2012). 10.1037/a0029393 22823292

[23] von Elm, E. et al. The Strengthening the Reporting of Observational Studies in Epidemiology (STROBE) statement: guidelines for reporting observational studies. *Lancet* **370**, 1453–1457 (2007). 18064739 10.1016/S0140-6736(07)61602-X

[24] Idler, E. L., Benyamini, Y., Self-Rated Health, Mortality & and A Review of Twenty-Seven Community Studies. *J. Health Soc. Behav.* **38**, 21–37 (1997). 9097506

[25] Johnson, F., Cooke, L., Croker, H. & Wardle, J. Changing perceptions of weight in Great Britain: comparison of two population surveys. *BMJ* 337 :a494, (2008). 10.1136/bmj.a494 PMC2500200 18617488

[26] Long, J. S. & Freese, J. *Regression Models for Categorical Dependent Variables Using Stata* (Stata, 2014).

[27] McCullagh, P. Regression Models for Ordinal Data. *J. Roy. Stat. Soc.: Ser. B (Methodol.)*. **42**, 109–127 (1980).

[28] Brant, R. Assessing Proportionality in the Proportional Odds Model for Ordinal Logistic Regression. *Biometrics* **46**, 1171–1178 (1990). 2085632

[29] Williams, R. Generalized ordered logit/partial proportional odds models for ordinal dependent variables. *Stata J.* **6**, 58–82 (2006).

[30] Silvapulle, M. J. & Sen, P. K. *Constrained Statistical Inference: Inequality, Order, and Shape Restrictions* (Wiley, 2005).

